# Thermal Oxidation Induces Lipid Peroxidation and Changes in the Physicochemical Properties and ***β***-Carotene Content of Arachis Oil

**DOI:** 10.1155/2015/806524

**Published:** 2015-03-03

**Authors:** Ayodeji Osmund Falade, Ganiyu Oboh

**Affiliations:** Functional Foods and Nutraceuticals Unit, Department of Biochemistry, Federal University of Technology, PMB 704, Akure 340252, Nigeria

## Abstract

This study sought to investigate the effect of thermal oxidation on the physicochemical properties, malondialdehyde, and *β*-carotene content of arachis oil. Pure arachis oil was heated for 20 mins with a corresponding temperature of 220°C. Thereafter, changes in the physicochemical properties (acid, iodine, and peroxide values) of the oil samples were determined. Subsequently, the level of lipid peroxidation was determined using change in malondialdehyde content. Then, the total carotenoid and *β*-carotene contents were evaluated using spectrophotometric method and high performance liquid chromatography, respectively. The results of the study revealed a significant increase (*P* < 0.05) in the acid and peroxide values and malondialdehyde concentration of the heated oil when compared with the fresh arachis oil. In contrast, a significant decrease (*P* < 0.05) was observed in the iodine value, total carotenoid, 13-cis-, 15-cis-, trans-, and 9-cis-*β*-carotene, and total *β*-carotene content of the heated oil. Hence, thermal oxidation induced lipid peroxidation and caused changes in the physicochemical properties and carotenoid contents of arachis oil, thereby reducing its nutritive value and health benefit. Therefore, cooking and frying with arachis oil for a long period might not be appropriate as this might lead to a loss of significant amount of the insignificant *β*-carotene in arachis oil.

## 1. Introduction

Arachis oil, also known as groundnut oil or peanut oil, is a mild tasting vegetable oil derived from peanuts or groundnuts. The oil is available in refined, unrefined, cold pressed, and roasted varieties, the latter with a strong peanut flavor and aroma, analogous to sesame oil [1]. Most highly refined arachis oils remove the peanut allergens and have been shown to be safe for the vast majority of peanut-allergic individuals [2].

Arachis oil is often used generally for cooking and is one of the most commonly consumed vegetable oils in Nigeria. Arachis oil has a high smoke point relative to many other cooking oils, so it is commonly used for frying foods [3]. Its major component fatty acids are oleic acid (46.8% as olein), linoleic acid (33.4% as linolein), and palmitic acid (10.0% as palmitin) [4]. The oil also contains some stearic acid, arachidic acid, arachidonic acid, behenic acid, lignoceric acid, and other fatty acids.

According to the USDA data, 100 g of arachis oil contains 17.7 g of saturated fat, 48.3 g of monounsaturated fat, and 33.4 g of polyunsaturated fat.

**Figure 1:**
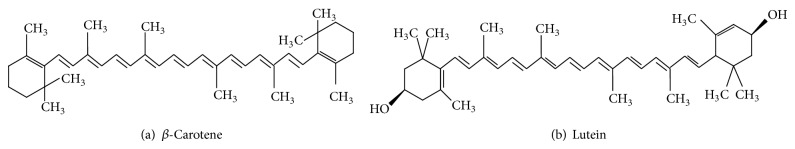
Structure of major pigments in arachis oil.

The pigments responsible for the arachis oil colour were postulated to be carotene [5] and xanthophylls [6]. Furthermore, previous work by Pattee et al. [7] revealed *β*-carotene and lutein (Figure 1) to be the major carotenoid pigments present in oil from arachis.

During frying process, various chemical reactions occur, such as thermal oxidation, hydrolysis, and polymerization, due to the exposure of the oil to high temperatures in the presence of air and moisture. As a result, cooking oil decomposes and forms volatile compounds and various monomers and polymers [8]. Several factors can affect the quality of cooking oil during heating, including ventilation, temperature, heating duration, the type of oil, the saturation ratio of the oil, and the presence of a catalyst/antioxidant [9].

**Figure 2:**
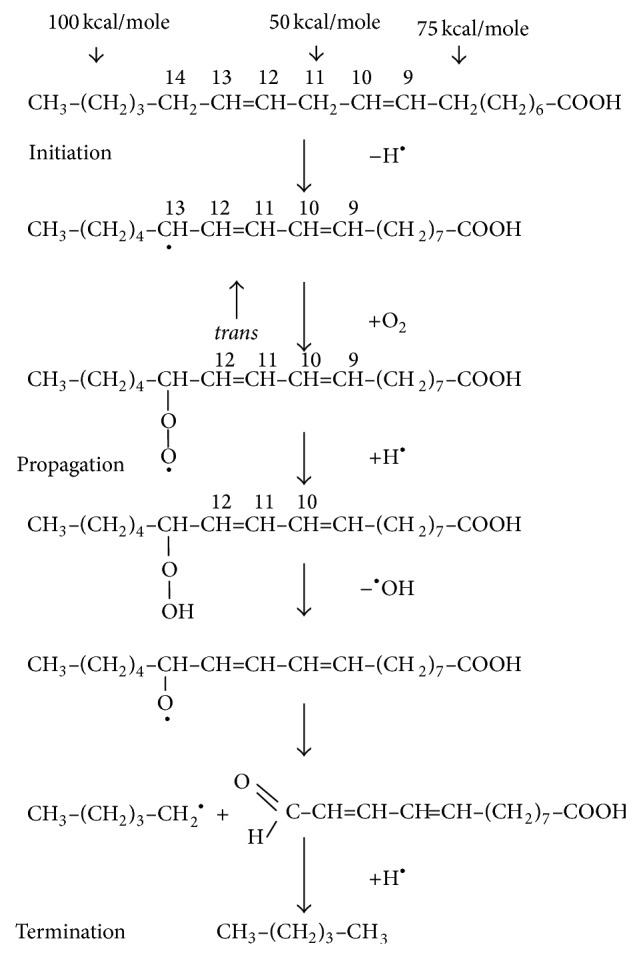
The initiation, propagation, and termination of thermal oxidation of oil. Source: Choe and Min [10].

The chemical mechanism of thermal oxidation is principally the same as the autoxidation mechanism. The thermal oxidation rate is faster than the autoxidation [10], but specific and detailed scientific information and comparisons of oxidation rates between thermal oxidation and autoxidation are not available. The mechanism of thermal oxidation involves the initiation, propagation, and termination of the reaction as shown in Figure 2.

Nonradical singlet state oil does not react with triplet state diradical oxygen due to the spin barrier. Ordinary oxygen in air is a diradical compound. Radical oxygen requires radical oil for the oxidation of oil. Therefore oil should be in a radical state to react with radical oxygen for oil oxidation reaction [10]. The hydrogen with the weakest bond on the carbon of oil will be removed first to become radical. The energy required to break carbon-hydrogen bond on the carbon 11 of linoleic acid is 50 kcal/mole [11]. The double bonds at carbon 9 and carbon 12 decrease the carbon-hydrogen bond at carbon 11 by withdrawing electrons. The carbon-hydrogen bond on carbon 8 or 11, which is *α* to the double bond of oleic acid, is about 75 kcal/mole. The carbon-hydrogen bond on the saturated carbon without any double bond next to it is approximately 100 kcal/mol [11]. The various strengths of hydrogen-carbon bond of fatty acids explain the differences of oxidation rates of stearic, oleic, linoleic, and linoleic acids during thermal oxidation or autoxidation [10]. The weakest carbon-hydrogen bond of linoleic acid is the one on carbon 11 and the hydrogen on carbon 11 will be removed first to form a radical at carbon 11 (Figure 2). The radical at carbon 11 will be rearranged to form conjugated pentadienyl radical at carbon 9 or carbon 13 with trans double bond as shown in Figure 2. Heat, light, metals, and reactive oxygen species facilitate the radical formation of oil [10].

Previous studies have shown that thermal oxidation has a deteriorative effect on dietary oils [12–15]. Nevertheless, vegetable oils are thermally oxidized during various food preparations to increase their palatability and this has been a usual domestic practice in most Nigerian homes [12]. Despite this common practice, there is limited information on the impact of heat treatment on the quality and nutritional value of arachis oil. Therefore this study sought to investigate the effect of thermal oxidation on the physicochemical properties and *β*-carotene content of arachis oil.

## 2. Materials and Methods

### 2.1. Materials

Chemicals and reagents used such as malondialdehyde-tetrabutylammonium salt (standard MDA), thiobarbituric acid (TBA), and trichloroacetic acid (TCA) were sourced from Sigma-Aldrich, Chemie GmbH (Steinheim, Germany); ethanol, methanol, hexane, dichloroethane, carbon tetrachloride, chloroform, glacial acetic acid, hydrochloric acid, and diethyl ether were sourced from BDH Chemicals Ltd. (Poole, England). Unless stated otherwise, all other chemicals are of analytical grades while the water was glass distilled.

### 2.2. Sample Collection and Preparation

Pure arachis oil (3 litres) with the brand name “grand pure groundnut oil” was sourced from the main market in Akure, Nigeria. Then 250 mL of the oil was heated {1000 W electric hot plate (Guangzhou D.G.H. Electrical Appliances Co., Ltd., Guangdong, China)} in a stainless steel frying pan for 20 mins and the corresponding temperature at that time was 220°C. Equal aliquot (100 mL) of the heated and fresh arachis oil was then withdrawn and kept in airtight amber bottles at room temperature prior to analysis.

### 2.3. Determination of Acid Value (AV)

The acid value was determined by modified titre metric method of Pearson [16]. Briefly, 1 g of the oil sample was dissolved in the mixed neutral solvent containing equal volumes of diethyl ether and ethanol; the indicator (1% phenolphthalein solution) was added and titrated against aqueous 0.1 M NaOH. The acid value was subsequently calculated.

### 2.4. Determination of Iodine Value (IV)

The iodine value was determined according to Wijs' method of Pearson [16]. Briefly, 1 g of the oil sample was dissolved in 2 mL of carbon tetrachloride. Thereafter, 4 mL of Wijs' solution was added and allowed to stand in the dark for 30 mins. Subsequently, 3 mL of 10% potassium iodide solution and 20 mL of water were added, mixed, and titrated against 0.1 M sodium thiosulphate solution using starch solution as an indicator. The iodine value was subsequently calculated.

### 2.5. Determination of Peroxide Value (PV)

The peroxide value was evaluated according to the method of AOAC [17] with slight modification. Briefly, 1 g of potassium iodide and 20 mL of solvent mixture (2 volumes of glacial acetic acid + 1 volume of chloroform) were added to 1 g of the oil sample in a clean dry boiling tube; the tube was placed in boiling water and allowed to boil vigorously for 30 s. Thereafter the content was then poured into a flask containing 20 mL of 5% potassium iodide solution and the tube was washed out with 25 mL water and titrated against 2 mM sodium thiosulphate solution using starch as an indicator. The peroxide value was subsequently calculated.

### 2.6. Lipid Peroxidation Assay

The malondialdehyde (MDA) content of the oil was measured as a product of lipid peroxidation using the modified method of Ohkawa et al. [18]. Briefly, 50 *μ*L of the oil sample was made up to 300 *μ*L with ethanol; thereafter, 300 *μ*L of 8.1% sodium dodecyl sulphate, 500 *μ*L of acetic acid/HCl buffer with pH 3.4 (Mettler Toledo Digital pH meter), and thiobarbituric acid (TBA) were added. The mixture was then incubated at 100°C for 1 h. Later the absorbance of the reacting mixture was taken at 532 nm. Subsequently, the MDA content was calculated using a MDA standard curve.

### 2.7. Determination of Total Carotenoid Content

The total carotenoid content was determined using a modified method of Spirulina Pacifica Technical Bulletin #003b [19]. Briefly, 4 mL diethyl ether was added to 0.5 mL of the oil sample in a 10 mL graduated centrifuge tube; this was subsequently saponified with 0.5 mL saturated potassium hydroxide (KOH) in distilled water in the dark for 30 mins. Thereafter, 5 mL of distilled water was added and centrifuged at 801 ×g for 3 mins. The volume of the ether layer was measured and its absorbance was taken at 450 nm, using diethyl ether as the blank. The absolute value of the total carotenoid was subsequently calculated.

### 2.8. High Performance Liquid Chromatography Analysis

The extraction and high performance liquid chromatography analysis were carried out using the modified method of Wills et al. [20]. The oil samples were dried down using TurboVap LV concentrator under nitrogen gas. The dried samples were redissolved with 60 mL ethanol containing 0.1% butylated hydroxytoluene (BHT) and mixed by vortexing. The mixture then underwent precipitation for 5 mins in the water bath at 85°C. Potassium hydroxide (500 *μ*L, 80% w/v) was added to the mixture to saponify the oil. Samples were vortexed and placed in a water bath (85°C) for 5 mins and vortexed again and returned to the water bath for additional 5 mins. Upon removal, they were immediately placed in an ice bath where 3 mL of cold deionized water was added. Carotenoids were separated three times with addition of 3 mL of hexane, vortexed, and then centrifuged at 1200 ×g for 30 s. The combined hexane fractions were washed with deionized water four times, vortexed, and centrifuged for 30 mins at 1200 ×g. The hexane fractions were dried down in a concentrator under nitrogen gas. Samples were reconstituted in methanol/dichloroethane (1 mL, 50 : 50 v/v); then 20 *μ*L and 100 *μ*L (high/low concentrations) were injected into the HPLC machine. Gradient elution was performed at 1 mL/min. A Waters HPLC system (Waters corporation, Milford, MA) consisting of a guard column, C30 YMC carotenoid column (4.6 × 250 mm, 3 *μ*L), Waters 626 binary HPLC pump, 717 autosampler, and a 2996 photodiode array detector was used for carotenoids quantification.

### 2.9. Data Analysis

Results of three replicates were pooled and expressed as mean ± standard deviation (STD). The Student* t*-test and the least significance difference (LSD) were carried out [21]. Significance was accepted at *P* ≤ 0.05.

## 3. Results and Discussion

**Figure 3:**
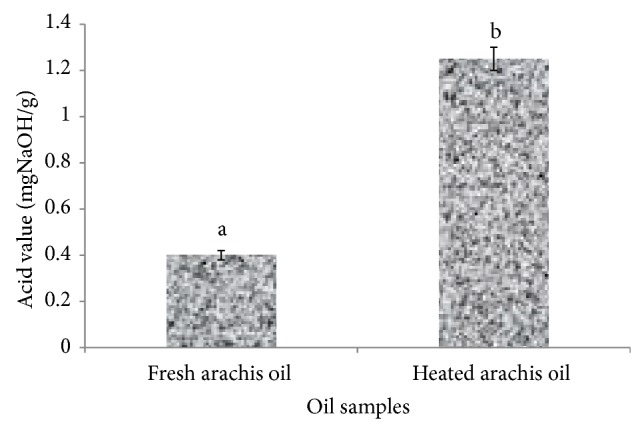
Effect of thermal oxidation on acid value of arachis oil. Acid values are expressed as mean ± standard deviation (*n* = 3). Bars with different alphabets are significantly different (*P* < 0.05).

Oxidation of edible oils occurs when monounsaturated fats (MUFA) and polyunsaturated fats (PUFA), which are mainly glycerol-bound, react with atmospheric O_2_. Primary oxidation products, hydroperoxides, are formed through different chemical mechanisms [22]. The hydroperoxides will further break down into secondary oxidation products (aldehydes, ketones, alkenals, etc.). Several of them possess toxic properties. Eventually, tertiary oxidation products (short chain free fatty acids) may be formed. These oxidation reactions may be accelerated by the presence of metals and exposure to heat and light [22]. Thermal oxidation of edible oils is a common practice in most homes in Nigeria. However, there is limited information on the impact of this domestic practice on the quality and nutritional value of arachis oil and hence the need for this study. The degree of oxidation of the oil was monitored by evaluating the physicochemical properties (acid, iodine, and peroxide values and malondialdehyde concentration) of the heated arachis oil. As shown in Figure 3 there was a significant (*P* < 0.05) increase in the acid value (AV) of the heated arachis oil (1.25 ± 0.05 mg NaOH/g) when compared with the fresh arachis oil (0.40 ± 0.02 mg NaOH/g). This result was in agreement with the findings of Khan and Nawaz [23] who reported that AV increased with increasing frying time during frying of chicken pieces with canola oil but was contrary to our previous work [12] where there was no significant difference in the AV of thermoxidized palm oil at different time interval. The discrepancies observed might be due to the level of saturation of the different oils investigated. However, the increase in AV observed in the heated arachis oil may be a result of thermal oxidative cleavage of triglycerides leading to the formation of free fatty acids (FFAs) in the oil. Free fatty acids and their oxidized compounds produce off-flavour [10] in edible oils. The higher the AV, the higher the deterioration or the rancidity of the oil.

**Figure 4:**
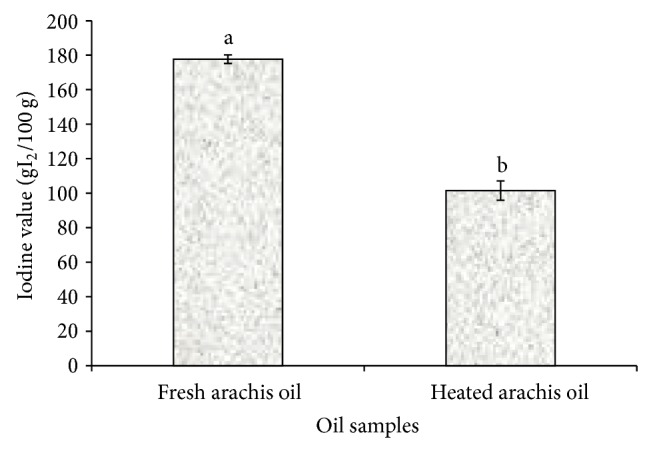
Effect of thermal oxidation on iodine value of arachis oil. Iodine values are expressed as mean ± standard deviation (*n* = 3). Bars with different alphabets are significantly different (*P* < 0.05).

**Figure 5:**
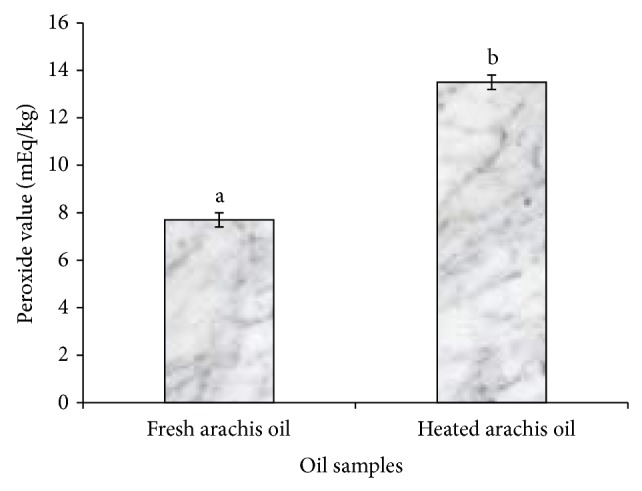
Effect of thermal oxidation on peroxide value of arachis oil. Peroxide values are expressed as mean ± standard deviation (*n* = 3). Bars with different alphabets are significantly different (*P* < 0.05).

In contrast, Figure 4 revealed a significant (*P* < 0.05) decrease in the iodine value (IV) of the heated arachis oil (101.52 ± 2.50 g I_2_/100 g) when compared with the fresh arachis oil (177.66 ± 5.60 g I_2_/100 g). This finding agreed with the work of Ayoola and Adeyeye [24], where a decrease in the iodine value was observed in groundnut oil after roasting. IV measures the degree of unsaturation of fatty acids; a decrease in IV as observed in this study indicates an increase in the degree of saturation of fatty acids which is a risk factor for hypercholesterolemia and some types of cardiovascular diseases [25]. This finding might be attributed to the fact that arachis oil contains more unsaturated fatty acids which are less stable and more readily broken down by heat. The repeated heating of edible oils that are rich in polyunsaturated fatty acids as in arachis oil has been reported to increase the formation of toxic compounds, which are associated with an increased risk of hypertension [26]. Furthermore, thermal oxidation caused a significant (*P* < 0.05) increase in the peroxide value (PV) of heated arachis oil (13.50 ± 0.30 mEq/Kg) when compared with the fresh (7.70 ± 0.30 mEq/Kg) arachis oil (Figure 5). This result is in close agreement with the recent work of Oboh et al. [12], where the peroxide value of thermoxidized palm oil increased significantly with heating (0 min: 3.00 mEqKg^−1^; 10 mins: 13.00 mEqKg^−1^). This finding also suggests that heating arachis oil for about 20 mins significantly (*P* < 0.05) increased the peroxide value above the standard PV (10 mEq/Kg) recommended for edible oil [27] which is indicative of deterioration of the oil. The increased PV observed in this study might be a result of formation and accumulation of peroxides induced by heating. The primary products of oxidation are hydroperoxides which then undergo further degradation to a variety of secondary decomposition products [28].

**Figure 6:**
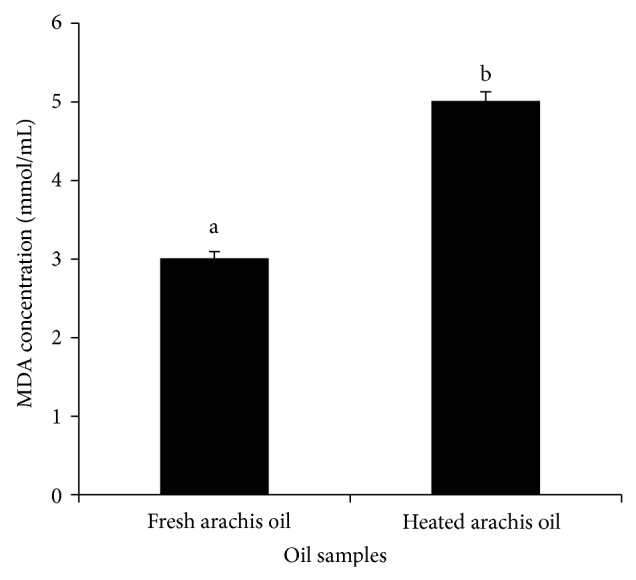
Effect of thermal oxidation on the MDA content of arachis oil. MDA contents are expressed as mean ± standard deviation (*n* = 3). Bars with different alphabets are significantly different (*P* < 0.05).

Similarly, the concentration of malondialdehyde (MDA), one of the well-known secondary products of lipid oxidation, increased significantly (*P* < 0.05) in the heated arachis oil (5.01 mmol/mL) when compared with the fresh (3.01 mmol/mL) arachis oil (Figure 6). This finding, which is in agreement with our previous work [12], might be attributed to the breakdown of peroxides to carbonyl and aldehyde compounds such as MDA, inducible by heating or high temperature [29]. The increased MDA concentration in this study suggests induction of lipid peroxidation. MDA is one of the many reactive electrophile species that cause oxidative stress in cells and form advanced glycation end-products (AGEs) [30] which have been implicated in some degenerative diseases such as cancer, diabetes mellitus, and kidney dysfunction [12]. MDA is able to form adducts with free amino acids and many more with proteins and this may induce profound alteration in their biochemical properties.

**Figure 7:**
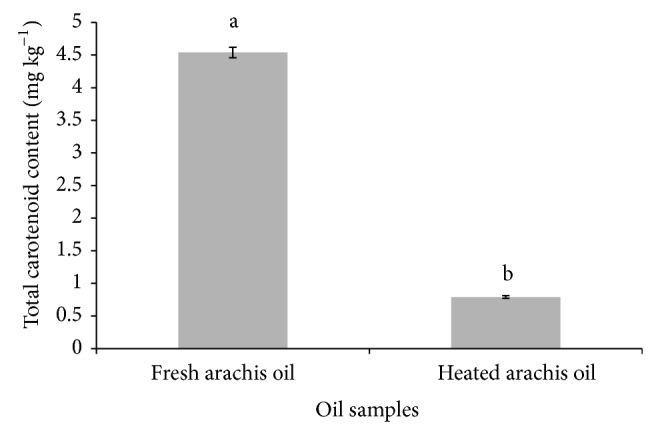
Effect of thermal oxidation on total carotenoid content of arachis oil. Total carotenoid contents are expressed as mean ± standard deviation (*n* = 3). Bars with different alphabets are significantly different (*P* < 0.05).

Furthermore, the result of the total carotenoid content as presented in Figure 7 showed a significant (*P* < 0.05) decrease in the total carotenoid content of the heated arachis oil (0.79 ± 0.01 mgKg^−1^) when compared with the fresh arachis oil (4.54 ± 0.02 mgKg^−1^). The total carotenoid content of the heated arachis oil was 17.4% of the total carotenoid content of the fresh arachis oil. This suggests that almost 82.6% of the carotenoid has been lost by thermal oxidation.

**Figure 8:**
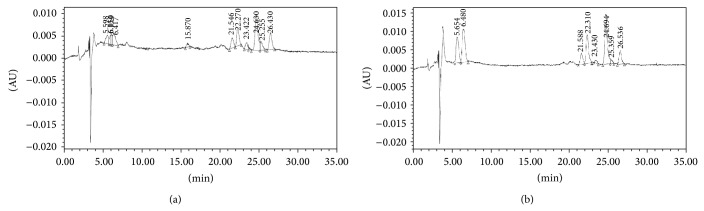
(a) Chromatogram of *β*-carotene of fresh arachis oil. (b) Chromatogram of *β*-carotene of heated arachis oil.

The high performance liquid chromatography analysis revealed the *β*-carotene profile in the arachis oil samples as affected by thermal oxidation (Figures 8(a) and 8(b), Table 1). The results revealed the presence of the following isomers of *β*-carotene in the oil samples: 13-cis; 15-*cis*;* trans*; and 9-*cis*. Nevertheless, the concentration of *β*-carotene decreased significantly (*P* < 0.05) in the heated arachis oil (13-*cis*: 0.07 *μ*g/g; 15-*cis*: 0.04 *μ*g/g;* trans*: 0.09 *μ*g/g; 9-*cis*: 0.05 *μ*g/g; total *β*-carotene: 0.25 *μ*g/g) when compared with the fresh arachis oil (13-*cis*: 0.11 *μ*g/g; 15-*cis*: 0.05 *μ*g/g;* trans*: 0.25 *μ*g/g; 9-*cis*: 0.09 *μ*g/g; total *β*-carotene: 0.50 *μ*g/g). This finding suggests that, at 20 mins of heating, about 50% of the total *β*-carotene in the oil was lost. The decrease in the carotenoid content observed in this study agreed with the report of Sulaeman et al. [31] on carrot chips deep fried in canola oil and partially hydrogenated soybean oil and our recent work, where thermal oxidation reduced carotenoid content in palm oil [12]. This finding might be attributed to the fact that carotenoid pigments are made up of a system of conjugated double bonds which are vulnerable to heat [12, 32]. The low concentration of *β*-carotene observed in this study agreed with the earlier claim of Pattee et al. [7] of insignificant total carotenoid concentration of the oil from fully matured peanuts. However, this study suggests that thermal oxidation causes a significant decrease in the insignificant concentration of *β*-carotene in arachis oil.

## 4. Conclusion

Thermal oxidation induced lipid peroxidation and caused changes in the physicochemical properties and carotenoid contents of arachis oil, thereby reducing its nutritive value and health benefit. Therefore, cooking and frying with arachis oil for a long period might not be appropriate as this might lead to a loss of significant amount of the insignificant *β*-carotene in arachis oil.

## Figures and Tables

**Table 1:** Effect of thermal oxidation on the concentration of *β*-carotene in arachis oil.

Oil sample	*β* carotene concentration (*μ*g/g)				Total *β*-carotene
	13-cis	15-cis	trans	9-cis	
Fresh arachis oil	0.16	0.05	0.25	0.09	0.50
Heated arachis oil	0.07	0.04	0.09	0.05	0.25
